# Soil Inoculation Alters Leaf Metabolic Profiles in Genetically Identical Plants

**DOI:** 10.1007/s10886-020-01156-8

**Published:** 2020-02-05

**Authors:** Martine Huberty, Beverly Martis, Jorian van Kampen, Young Hae Choi, Klaas Vrieling, Peter G. L. Klinkhamer, T. Martijn Bezemer

**Affiliations:** 1 Department of Terrestrial Ecology, Netherlands Institute of Ecology, Wageningen, The Netherlands; 2 Plant Ecology and Phytochemistry, Institute of Biology, Leiden University, Leiden, The Netherlands; 3 Natural Products Laboratory, Institute of Biology, Leiden University, Leiden, The Netherlands; 4 College of Pharmacy, Kyung Hee University, Seoul, Republic of Korea

**Keywords:** Ecometabolomics, Aboveground-belowground interactions, Plant-soil interaction, Phytobiome, Nuclear magnetic resonance spectroscopy, *Jacobaea vulgaris*

## Abstract

Abiotic and biotic properties of soil can influence growth and chemical composition of plants. Although it is well-known that soil microbial composition can vary greatly spatially, how this variation affects plant chemical composition is poorly understood. We grew genetically identical *Jacobaea vulgaris* in sterilized soil inoculated with live soil collected from four natural grasslands and in 100% sterilized soil. Within each grassland we sampled eight plots, totalling 32 different inocula. Two samples per plot were collected, leading to three levels of spatial variation: within plot, between and within grasslands. The leaf metabolome was analysed with ^1^H Nuclear magnetic resonance spectroscopy (NMR) to investigate if inoculation altered the metabolome of plants and how this varied between and within grasslands. Inoculation led to changes in metabolomics profiles of *J. vulgaris* in two out of four sites. Plants grown in sterilized and inoculated soils differed in concentrations of malic acid, tyrosine, trehalose and two pyrrolizidine alkaloids (PA). Metabolomes of plants grown in inoculated soils from different sites varied in glucose, malic acid, trehalose, tyrosine and in one PA. The metabolome of plants grown in soils with inocula from the same site was more similar than with inocula from distant sites. We show that soil influences leaf metabolomes. Performance of aboveground insects often depends on chemical composition of plants. Hence our results imply that soil microbial communities, via affecting aboveground plant metabolomes, can impact aboveground plant-insect food chains but that it is difficult to make general predictions due to spatial variation in soil microbiomes.

**Electronic supplementary material:**

The online version of this article (10.1007/s10886-020-01156-8) contains supplementary material, which is available to authorized users.

## Introduction

Plants are sessile and this limits their capacity to escape unfavourable conditions in their surroundings. As plants cannot escape from exposure to organisms such as pathogens, herbivores and symbionts aboveground as well as belowground, they produce a vast array of chemical compounds to protect themselves. However, such compounds can also be used by these organisms as e.g. feeding stimulants or for host recognition (Macel 2011). Therefore, chemical variation among plants is a key factor in understanding interactions between plants and their environment (Dyer et al. 2018). The potential threats to plants vary spatially, both between and within different sites. This raises the question if plants can adjust their chemical composition according to the pests and pathogens they are confronted with at a local scale. For insect herbivores there is ample evidence that this is indeed the case (Kleine and Müller 2011). The soil microbial community also varies greatly between sites and even spatially within a single location. It is well-known that variation in soil such as changes in the microbial community affects the growth of plants and the composition of plant communities (Van der Putten et al. 2016; Wang et al. 2019a,b), how such changes in the soil, that we expect to be the result of soil inoculation, affect the chemical composition of plants is less well understood (Bezemer et al. 2005).

Several studies have shown that the foliar chemistry of plants may vary depending on the soil in which they grow (e.g. Kos et al. 2015a; Ristok et al. 2019; Zhu et al. 2018). This can be attributed to differences in abiotic properties of soils, such as nutrient or water availability, but also to differences in soil biota, for example, the presence of arbuscular mycorrhizal fungi (AMF) or beneficial rhizosphere bacteria in the soil (Schweiger et al. 2014; Zhou et al. 2018). Hill et al. (2018) recently showed that 33 compounds in the root metabolome of *Jacobaea vulgaris* changed in plants after exposure to the arbuscular mycorrhizal fungus (AMF) *Rhizophagus irregularis*, even though no compounds changed in the leaf metabolome. These studies examined the effect of one isolated group of soil organisms on the chemical composition of plants. Other studies focused on the total microbial community: PAs and amino acid composition and concentration in *J.vulgaris*, for example, depend on the microbial community of the soil in which the plant grows (Kos et al. 2015b; Kostenko et al. 2012; Wang et al. 2019b). Recently, Ristok et al. (2019) reported that changes in plant species richness lead to soil biotic legacies that subsequently elicit changes in the metabolomes of later growing plants. In most of these studies, test plants were grown in sterilized bulk soil that was inoculated with a small portion of live soil collected from potted plants or monocultures, or with watery extracts of live soil from potted plants. In this way all plants are grown in soil with comparable abiotic conditions but with different soil microbiomes (Wang et al. 2019b).

Soil biota can influence the plant metabolome either directly by triggering a response in the plant, such as induced systemic resistance (Van de Mortel et al. 2012), or indirectly by influencing the growth of the plant, since the composition of many plant compounds is related to the growth of the plant and associated characteristics such as the shoot/ root ratio. For example, *J. vulgaris* plants with lower biomass often have higher concentrations of pyrrolizidine alkaloids (PAs) than plants with higher biomass, because the concentration of these toxic PAs is diluted in larger plants (Hol 2011).

Microbiomes in the soil are altered by both biotic and abiotic factors of the soil and are highly dynamic (O’Brien et al. 2016). Hence, even closely located sampling points can harbour soil microbiomes that differ greatly as the abiotic and biotic properties of the soil might differ even at a scale of millimetres or centimetres (Ettema and Wardle 2002; Fierer 2017). To what extent those potenial spatial differences in microbiome composition in the soil influence the chemistry of plants growing in those soils is poorly understood. In this study we used *J. vulgaris*, a monocarpic perennial herb, native to Europe and Asia and invasive in North America, Australia and New Zealand (Bain 1991). *J. vulgaris* can grow in a broad range of soils and in a range of diverse habitats, such as sand dunes, woodlands and grasslands (Bezemer et al. 2006). Pyrrolizidine alkaloids are one of the major groups of secondary metabolites in *Jacobaea* species and are known to influence interactions of the plants with insects (Macel 2011). Several studies in which *J. vulgaris* was grown in sterilized soil inoculated with soil collected from different locations within a single grassland show that plant biomass varies depending on the soil sample that was used as inoculum (Kos et al. 2013; Bezemer et al. 2005). As the concentration of PAs in this species is linked to biomass (Hol 2011) this suggests that this group of compounds may also vary among those spatially collected soil inocula.

Up to now, most studies that examined the effects of soil inoculation on plant chemical compounds used targeted approaches (e.g. Zhu et al. 2018; Kos et al. 2015). The metabolome of a plant, however, is highly diverse, and changes in one or a few specific compounds or groups of compounds are unlikely to represent a realistic picture of the metabolic changes that occur within the plant. Therefore, untargeted metabolomic approaches are preferred to investigate the chemical response of plants to soil inoculation. In this study, we inoculated sterilised soil with soil collected from four natural grasslands in The Netherlands. Within each grassland we collected soils from different locations at fixed distances and collected two samples within each plot so that there were three different spatial scales in our experimental design (plot, within sites and between sites). These soils presumably differed in microbiome composition, but we did not measure that in this present study. We grew *J.vulgaris* in sterilized bulk soil inoculated with the different soils and used genetically identical *J. vulgaris* plants. We measured the leaf metabolome using ^1^H Nuclear magnetic resonance spectroscopy (^1^H-NMR), which enabled us to detect a large range of chemical compounds, including both primary and secondary metabolites as well as polar and non-polar metabolites.

We hypothesize that (i) the metabolic composition of *J. vulgaris* will vary between inoculated and uninoculated soils; that (ii) metabolomes will vary among the sites the soil was collected from; and (iii) that metabolomes of plants growing in inoculated soils collected from the same grassland will be more similar than when the inocula originate from different grasslands.

## Methods and Materials

Inoculation soil was collected in early March 2017 from four different natural grasslands in the Netherlands at sandy soils. Two sites A (N52 ^o^09.259′ E4 ^o^22.847′) and B (N52 ^o^09.770′ E4 ^o^23.520′) were natural grasslands near the Dutch coast, and two sites C (N52 ^o^01.613′ E5 ͦ48.379′) and D (N52 ^o^00.694′ E5 ^o^46.877′) were natural grasslands at the Veluwe area in the mainland of the Netherlands. All sites were nature areas on sandy soils that were formerly used for agricultural purposes. At each site, soil samples were collected along two transects that were laid out in a 100 by 200 m area in which no visible gradient in vegetation was observed. Each transect consisted of four plots (30 × 30 cm) at 0 m, 20 m 60 m and 100 m distance. The distance between the two transects was 200 m. Two soil cores were taken in each plot at 15 cm depth with a soil auger (⌀ 7 cm). Each sample was kept separate so that there were 16 samples per grassland. The samples were sieved individually through a sterilized sieve (1 cm) and stored at 4 °C.

### Bulk Soil

For sterilized bulk soil we collected 300 kg soil from a natural grassland at the Veluwe “De Mossel” (Ede, The Netherlands). This soil was sieved through a 1 cm sieve, homogenized and sterilised by γ-irradiation (> 25 KGray, Synergy Health, Ede, The Netherlands). The soil is a sandy loam soil (85% sand, 10% silt, 3% clay, 3% organic matter, pH 4.5, N total 1332 mg/kg; P plant available 4 mg/kg, K plant available 41 mg/kg, Mg plant available 55 mg/kg; S Total 208 mg/kg).

To preclude variation in the metabolome due to genetic differences we used tissue cultured plants in this study. In a climate room 200 *J. vulgaris* cuttings from a single genotype were asexually propagated in tissue culture using MS medium (Murashige and Skoog medium) with 100 mg/L benzylaminopurine (BAP) (16:8 h light:dark photoperiod, 20 °C). To produce roots the cuttings were grown in MS medium without BAP for 10 days. The genotype that was propagated was formerly collected from Meijendel (Wassenaar), The Netherlands.

### Experimental Phase

For each pot 45 g of (live) soil was mixed with 405 g sterilized bulk soil (1:9 ratio). Each mixture was prepared individually and homogenized in a new plastic bag. There were 64 (4 sites × 8 plots × 2 samples) mixtures of different soils. In addition, five pots were filled with 450 g sterilized soil and used as control giving a total of 69 pots. The pots were randomly placed in the climate room (16 h: 8 h light: dark photoperiod, 20 °C) and covered with plastic foil for 5 days to maintain humidity and allow the microbial community to establish before proceeding with planting.

The size of the 200 *J. vulgaris* plantlets was visually inspected and 69 similar sized (longest leaf ±4 cm) plantlets were selected and one plantlet was transplanted into each pot. Seedlings that emerged from the soil were removed every 2 days. The pots were placed in blocks in the climate room and the position of pots within each block was randomised once a week. To control the moisture of the soils and to account for potential differences in water usage depending on the inocula, the pots were individually reset to the same humidity (pot weight) twice per week. During other days, all plants received the same amount of water. Six weeks after planting shoots were clipped to determine biomass and used for metabolomics analyses. The leaves were immediately wrapped in aluminium foil and flash frozen in liquid nitrogen and stored at −80 °C until lyophilisation. Shoots were lyophilised for 72 h. To investigate if the nutrient content of the soils differed between the inocula, the soil from each pot was dried at 40 °C for a subset of 37 samples in the oven for soil chemical analysis (see Soil Chemical Analysis for details). The roots were carefully washed, dried and weight. After the lyophilisation shoot dryweight of each plant was determined. All lyophilised plant material was stored at room temperature in plastic bags with silica gel.

### Soil Chemical Analysis

Soil chemical analysis were conducted on a subset of the samples collected after the plants had grown in the soil. For the analysis we randomly selected soils from 4 of the 8 plots per grassland. Both replicates of each plot (4 grassland sites × 4 random plots × 2 replicates) were analysed as well as soil from five control pots. Oven-dried soil samples (40 °C) were sieved through a 2 mm sieve and 3 g of dry soil was added to 30 mL of 0.01 M CaCl_2_ and shaken for 2 h at 250 rpm. Soil samples were centrifuged for 5 min at 3000 rpm and 15 mL of the supernatant was filtered through a syringe filter (cellulose acetate membrane). 12.86 mL of this filtrate was vortexed, and Fe, K, Mg, P, S and Zn were measured the following day (ICP-OES, Thermo Scientific iCAP 6500 Duo). The remaining filtrate was used to measure NO_2_ + NO_3_ and NH_4_ on a QuAAtro Autoanalyzer (Seal analytical).

### Metabolomics ^1^H NMR Analysis

The extraction of the leaf samples was done following an adapted version of the protocol described by Kim et al. (2010). The lyophilised plant material was ground in a micro tube (1.5 ml) with one metal ball bearing and placed in a TissueLyser (Retsch Mixer Mill MM 400) for 3 min at 30 s^−1^. Then, 20 mg ± 1 mg was transferred to a 1.5 ml microtube and 300 μl CH_3_OH-d_4_ (Sigma, St Luis, MI, USA) followed by 300 μl KH_2_PO_4_- D_2_O buffer with 0.01% TSP was added to the weighed plant material. The samples were then sonicated for 10 min and were centrifuged at 13.000 ppm for 10 min. 250 μl of the supernatant was collected and transferred to an NMR tube (103.5 × 3 mm, inside-ø 2.24 ± 0.05 mm).

^1^H NMR spectra were recorded on a Bruker AV-600 MHz NMR spectrometer (Bruker, Karlsruhe, Germany), operating at a frequency of 600.13 MHz. As an internal lock we used CH_3_OH-*d*_4._^1^H NMR spectra were recorded with pulse width (PW) = 30 ° (11.3 μs), Relaxation delay (RD) = 1.5 s and 128 scans with a total of 10 min and 26 s acquisition time with 0.16 Hz/point. A presaturation sequence was used to reduce the signal of H_2_O frequency during the recycle delay. FIDs were Fourier transformed by a line broadening of 0.3 Hz. Spectra were then manually baseline corrected, calibrated to TSP at 0.00 ppm, and phased in TOPSIN (v.3.0. Bruker). Then the data was bucketed with scaling to total intensity and a bucket width of 0.04 ppm in AMIX software (v. 3.9.12 Bruker BioSpin GmbH, Reinstetten, Germany). Bucketing or binning is commonly used in metabolomics to reduce the effect of small shifts of signals between samples (Kim et al. 2010). Residual signals from solvents in regions between 4.70–4.90 ppm and 3.28–3.34 ppm were excluded. The pre-processing therefore leads to a data matrix with 246 buckets per sample. Each bucket contains the signals from the NMR within the range of 0.04 ppm and directly represents the molar level of a compound leading to a signal in this region of the NMR. In ^1^H-NMR all H atoms within one molecule lead to signals. Therefore, molecules consisting of more than one H atom lead to signals in several buckets. The chemical shift of the signal depends on the chemical environment of the H atom and is defined by the neighbouring atoms of the H atom. Furthermore, the neighbouring atoms influence the splitting pattern of a signal in the NMR. Here we used the chemical shift and the splitting pattern to identify the compounds in the NMR and compared them to an internal database (for details see Kim et al. 2010). The compounds were putatively identified. PAs could not be specified in depth with NMR and are therefore only referred to as PAs.

### Data Analysis

If not mentioned otherwise all analyses were performed in R Studio (RStudio Team, 2016) using the package ‘vegan’ (Oksanen et al. 2018) and the function and the function ‘pairwise.Adonis’(Martinez 2017). Volcano plots were made using Metaboanalyst (Chong et al. 2018). Co-correspondence analysis (CoCA) was done in in CANOCO 5 (Šmilauer and Lepš 2014).

We visualised the foliar metabolome changes (intensity in buckets) due to inoculation by non-metric multi-dimensional scaling (NMDS) based on Bray-Curtis dissimilarities. NMDS is a method that uses a dissimilarity matrix to produce an ordination which represents the dissimilarities between objects in a low-dimensional space. We used Bray-Curtis dissimilarities as this method uses intensities of the measured signals rather than presence/absence data.

To examine if the different inocula varied in how they changed the metabolome of *J.vulgaris* we conducted a permutational analysis of variance (PERMANOVA) based on Bray Curtis dissimilarities. Permutations were set to 999. For this analysis the data of the plants grown in 100% sterilized soil was removed. We conducted a PERMANOVA with the fixed factor “site” and the covariate “shoot dry biomass”. With a second PERMANOVA we analysed if changes in the metabolome are linked to root biomass by including the factors “site” and the covariate “root dry biomass”. To investigate the biomass effect on the metabolome, we conducted a Pearson correlation analysis of the intensity of signals in each bucket with the shoot biomass of the plants. *P* values were then corrected for multiple testing by false discovery rate (FDR) (Benjamini and Hochberg 1995).

We used variance partitioning (Multivariate redundancy analysis RDA) using the function “varpart” to disentangle the effects of site and shoot biomass on the metabolome of *J. vulgaris*. For this the data of the plants grown on 100% sterile soil was removed from the dataset. We tested the significance of the marginal and conditional effects of both predictors with a Monte Carlo permutation test (999 permutations).

To investigate if inoculation influenced the chemical diversity of the plants growing in the soils, we calculated the Shannon evenness of the plant metabolomes. A one-way analysis of variance (ANOVA) with a Dunnett post hoc test was conducted to compare the Shannon evenness of plants grown in pots inoculated with soil from different sites (four levels) and plants grown in sterilized soil (1 level).

To analyse site-specific effects on the Shannon evenness an ANOVA with fixed factor “site” and biomass of the shoot as a covariate was conducted. For this analysis the data of the plants grown in 100% sterilised soil was excluded.

To visualise the metabolomic differences of plants grown in sterilised soil and inoculated soil we used volcano plots. For the volcano plots the log_2_ fold-change between plants grown in sterilized soil and inoculated soil was calculated per site. For each bucket an ANOVA was used to compare plants grown in sterilized and inoculated soil and the *P*-values were log_10_-transformed. Then the log_2_ fold-change was plotted against the log P-values. This enabled us to visualise which signals in the NMR differed most significantly (fold-change) between plants grown in inoculated and sterilized soil.

To examine the effect of different spatial scales we used the Bray-Curtis dissimilarity for all pairs of two plants growing in soil from the same plot at the same site (plot scale, eight per site); one random pair of the same plot with a plant growing in soil from different plots at the same site (site scale, 16 per site) and random pairs of each plant with plants growing in plots from different sites (large scale, 16 per site). The Bray Curtis dissimilarities were then analysed with ANOVA with as fixed factors scale (plot scale, site scale, large scale) and site (A, B, C, D).

Shoot biomass and root biomass of plants grown in pots inoculated with soil from the four different sites and plants grown in sterilized soil were analysed using one-way ANOVA followed by a Dunnett post hoc test. To compare the site-specific effects of the different soil inocula on the biomass of *J .vulgaris* plants, plants grown in 100% sterilized soil were removed from the dataset and with a new ANOVA the effect of the soil from different sites as well as the plots within each site was compared (plot nested within site). With a Tuckey post-hoc test treatments were compared to each other. All data was checked for homogeneity of variance and normal distribution of the residuals. Shoot biomass was square root-transformed to obtain normality of the residuals.

Soil parameters were analyzed using ANOVA with site as fixed factor. For the soil parameters the plot effects could not be accessed because we only measured soil parameters for a subset of samples from each site.

The relationship between soil characteristics and leaf metabolome composition, was analysed using a co-correspondence analysis (CoCA) in CANOCO 5 (Šmilauer and Lepš 2014) whereby the soil abiotic parameters where centred and standardized. A Monte Carlo permutation test with unrestricted permutations for all axes was done as described in Šmilauer and Lepš (2014).

A metabolic pathway of *S. vulgaris* was constructed with the help of KEGG reference pathways (Kanehisa and Goto 2000) and mean values for the intensity of the signals of buckets associated to the compounds displayed in the pathway were calculated and displayed in the metabolic pathway map.

## Results

**Fig. 1:**
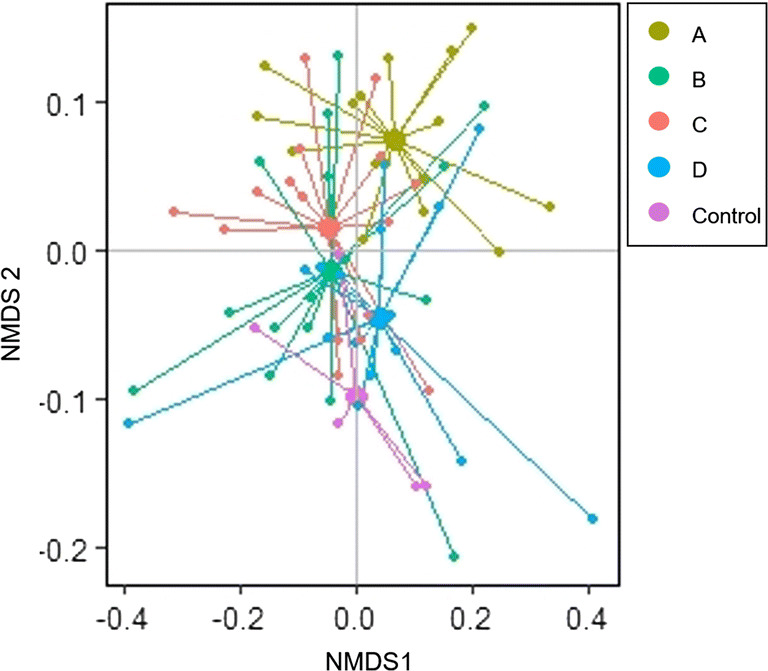
Non-metric multidimensional scaling (NMDS) plot of the metabolome of *Jacobaea vulgaris* grown in inoculated soil from different sites (A, B, C, D) and in 100% sterilized soil using Bray-Curtis dissimilarities. Shown are centroids (large circles) and individual samples (small circles) for each site and for the control. The stress is a measurement for the fit of the model and was 0.10

The composition of the leaf metabolomes of *J. vulgaris* varied significantly among the four sites from which the inocula originated (Fig. 1, Table 1) and was significantly related to shoot biomass (Table 1, Supplementary Fig. 1). In a PERMANOVA, site explained 13% of the variation in the leaf metabolome and shoot biomass 18% (Table 1). In a pairwise comparison the metabolome of plants grown in inoculum A was different from metabolomes of plants grown in the other soils (Supplementary Table 1, Table 2). Variance partitioning showed comparable results with 5% of the metabolome variation solely being explained by the different sites and 22% by shoot biomass (Table 2). All marginal and conditional effects of the predictors were significant. A PERMANOVA which included root biomass instead of shoot biomass showed that root biomass did not significantly explain variation in the metabolome (Supplementary Table 3).

**Table 1:** Results of permutational multivariate analysis of variance (PERMANOVA) testing the effect of inoculation with soil from four different sites (A, B, C, D) and shoot biomass on the metabolome of *Jacobaea vulgaris.* Presented are F-values with degrees of freedom (df), explained variance (R^2^) and P values. Permutations were set to 999. Significant factors are indicated in bold

	F-value	R^2^	P value
**Site**	F_(3,56)_ = 3.94	0.13	0.001
**Shoot biomass**	F_(1,56)_ = 16.80	0.18	0.001
Site * Shoot biomass	F_(3,56)_ = 2.09	0.07	0.035

**Table 2:** Variance partitioning (Multivariate redundancy analysis RDA) of the effect of site (A, B, C, D) and shoot biomass on the metabolome of *Jacobaea vulgaris*. Depicted are marginal (explanatory variable alone) and conditional effects. The conditional effects were calculated by using one factor as main factor and the other factor as covariable indicate by /. For each combination degrees of freedom (df) and adjusted R^2^ values and *P*-values from Monte Carlo permutation test (999 permutations) are depicted. Significant factors are indicated in bold

Factor	df	Adjusted R^2^	P-value
**Site**	4	0.09	0.001
**Shoot biomass**	1	0.26	0.012
**Site + Shoot biomass**	5	0.31	0.001
**Site/ Shoot biomass**	4	0.05	0.011
**Shoot biomass/Site**	1	0.22	0.001

^1^H signals in the metabolomes that differed between sites from which the soil inocula was collected from were glucose, malic acid, trehalose, tyrosine, unknown/unidentified PAs (PA A) and two other unknown compounds (Supplementary Fig. 2a). The intensity of multiple signals in the NMR depended on shoot biomass (Supplementary Fig. 2c, Fig. 3). Changes in the intensities of amino acids, sugars (mannitol, glucose, raffinose and other signals related to sugar compounds which could not be determined more precisely) were related to biomass. 80 out of the 96 significant correlations between signal intensity and biomass were negative, strongly showing the dilution effect. This effect was especially strong for amino acids, phenolic compounds and terpenoids (all negative) while the opposite was found for the sugars (with significant positive correlations) (Supplementary Fig. 3).

**Fig. 2:**
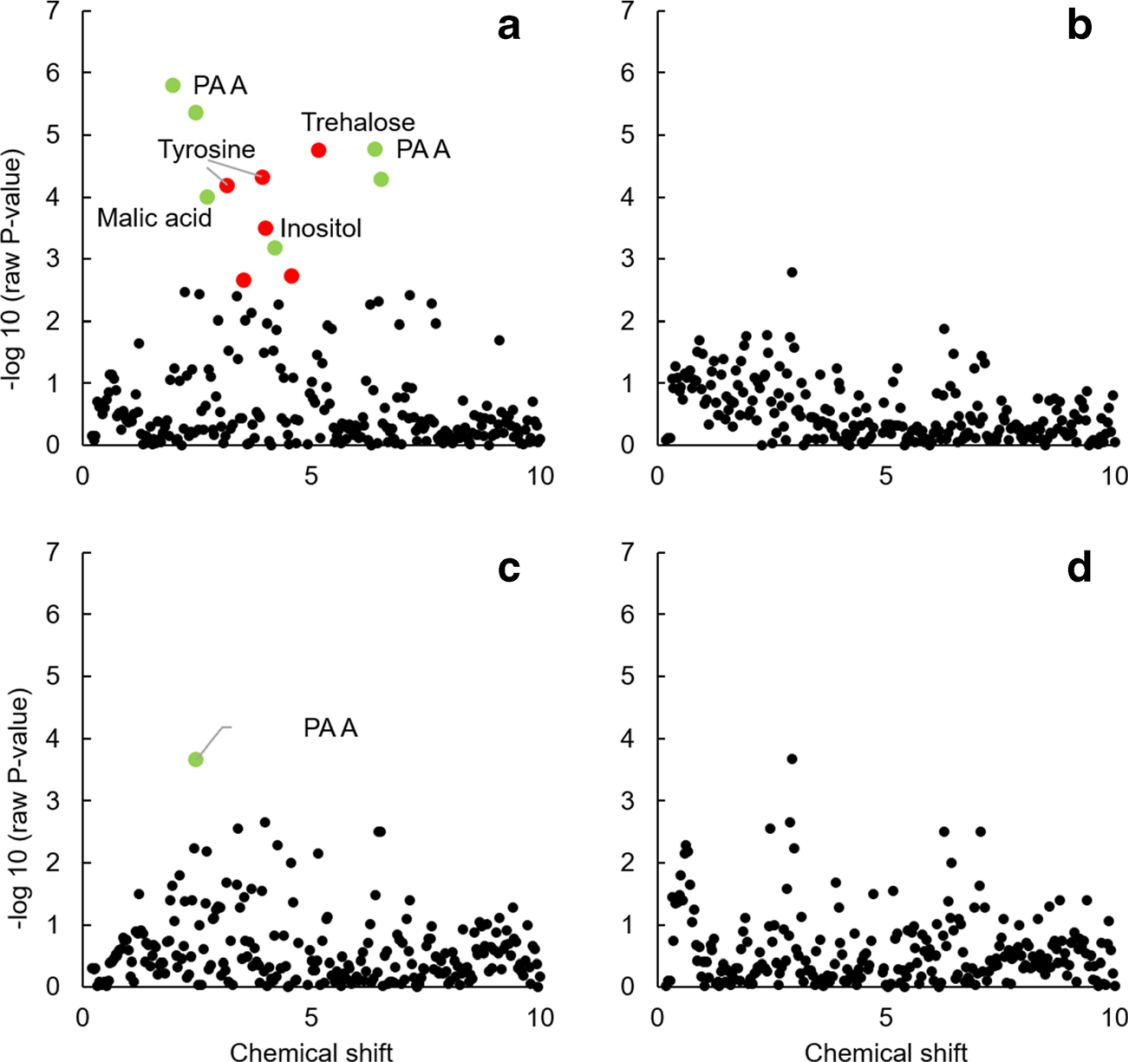
Negative logarithm of *P* values of a t-test testing for differences within each bucket (each chemical shift) in the intensity of the signals representing the metabolome of *Jacobaea vulgaris* grown in 100% sterilized soil and in soil inoculated with soil collected from site A, B, C and D. Red coloured dots represent buckets in which the signal showed a higher intensity and green dots buckets with a lower intensity in plants grown in inoculated soil than in 100% sterilized soil. For signals that showed a P value <0.05 after false discovery correction (FDR) identifications are indicated. Signals were tentatively associated to compounds: pyrrolizidine alkaloid A (PA A) 1.96, and 6.4 ppm, trehalose 5.16 ppm, tyrosine 3.16 and 3.92 ppm, malic acid 2.72 ppm, inositol 4.00 ppm. Coloured dots without a description are from signals which could not be assigned to a specific compound

We subsequently compared metabolomes of plants grown in inoculated soil with plants in sterilized soil for each site separately. The concentration of PA A was lower in plants grown in inoculated soils than in 100% sterilized soil for sites A, C and D (Supplementary Fig. 4). The concentrations of trehalose, tyrosine and inositol were significantly higher, and the concentration of PA A was lower in plants grown in inoculated soil than in 100% sterilized soil for site A. Malic acid was lower in plants grown in inoculated soil from site A than in plants grown in 100% sterilized soil. Overall, inoculation with soil from site A led to most changes in the metabolome (Fig. 2, Supplementary Fig,4, Table 2).

**Fig. 3:**
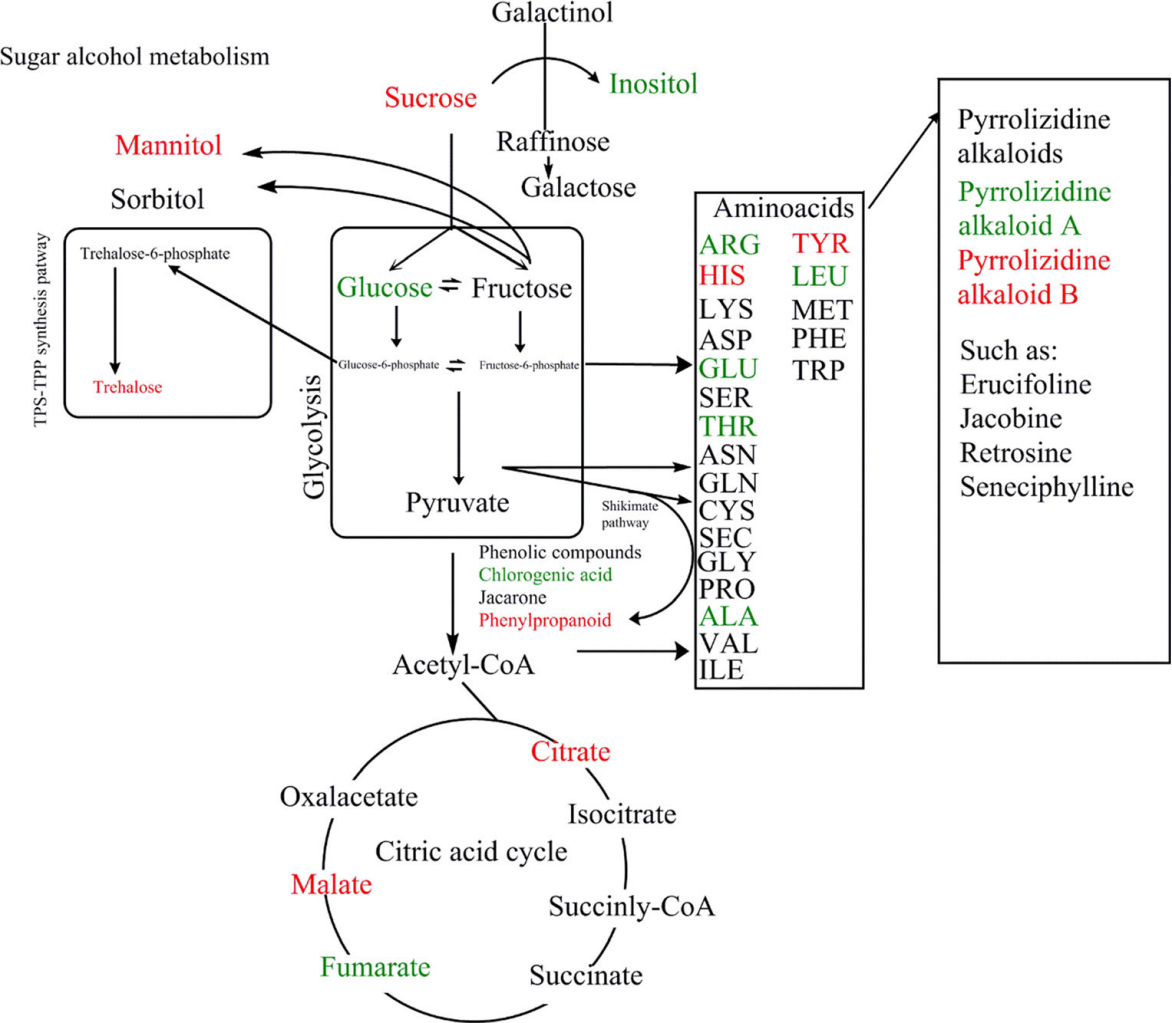
Metabolic pathway of *Jacobaea vulgaris* displaying changes in the pathway due to growth in inoculated or in 100% sterilized soil. Green font indicates higher and red font lower concentrations of compounds in plants grown in 100% sterilized soil than in inoculated soil. This pathway only depicts the main pathways; not all reactions and intermediates are depicted. The compounds displayed were associated to signals as follows: glucose 5.2 ppm, sucrose 6.60 ppm, citrate 2.52 ppm, malate 4.32 ppm, mannitol 3.84 ppm, inositol 4.04 ppm, malic acid 2.64 ppm, pyrrolizidine alkaloid A 6.42 ppm, pyrrolizidine alkaloid B 2.56 ppm, ARG 1.7 ppm, HIS 8.1 ppm, GLU 2.4 ppm, THR 1.3 ppm, ALA 1.5 ppm, TYR 3.16 ppm, LEU 0.9 ppm, fumarate 6.73 ppm, phenylpropanoid 6.45 ppm. Compounds presented in black could not be identified

Inoculation led to changes in the metabolome of *J.vulgaris* in various parts of the metabolic pathway (Fig. 3). Concentrations of certain amino acids (ARG, GLU, THR, ALA, LEU) were reduced in plants grown in sterilized soil while the concentrations of the amino acids TYR and HIS were higher in plants grown in sterilized soil. Compounds related to the sugar metabolism and tricarboxylic acid cycle (TCA) were also influenced by inoculation.

**Fig. 4:**
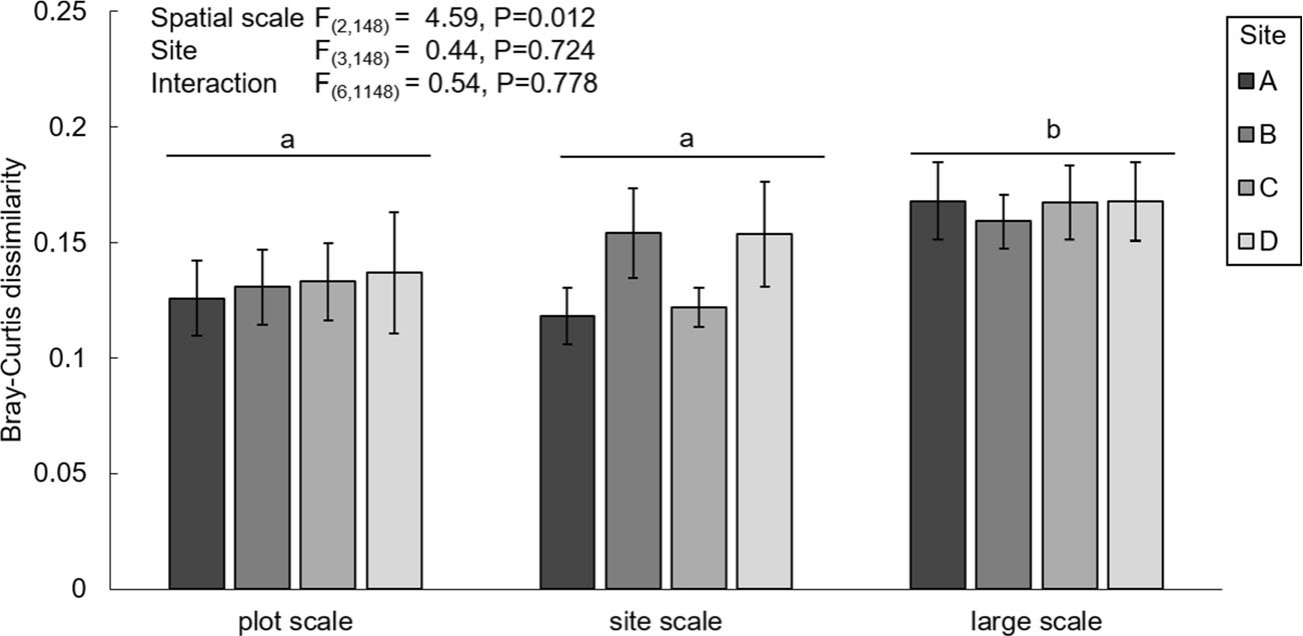
Mean Bray-Curtis dissimilarity (± SE) of the leaf metabolome of *Jacobaea vulgaris* plants grown in inoculated soil originating from the same plot (plot scale), from the same site but from different plots (site scale), or from different sites (large scale). The values for each site are presented in different shades of grey. Results from an analysis of variance (ANOVA) with fixed factors “spatial scale” (plot, site, large) and “site” are indicated in the left upper corner

The dissimilarity in metabolome composition did not vary significantly between sites (ANOVA: F_(3,148)_ = 0.44 *P* = 0.724). However, the Bray-Curtis dissimilarity differed between the different spatial scales (plot scale, site scale, large scale) (ANOVA: F_(2,148)_ = 4.59, *P* = 0.012) (Fig. 4). The Bray-Curtis similarity was on average highest when two samples were compared of plants grown with inocula collected from the same plot. The chemical diversity of the metabolome, measured as Shannon evenness did not differ significantly between sites (ANOVA: F_(3,59)_ = 2.31, *P* = 0.086) (Supplementary Fig. 5) but did depend on the shoot biomass of the plants (ANOVA: F_(1,59)_ = 17.91, *P* < 0.001).

**Fig. 5:**
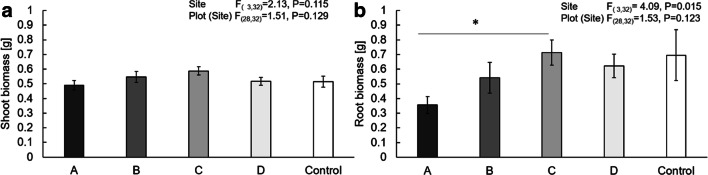
Mean dry biomass ±SE of a) shoots and b) roots of *Jacobaea vulgaris* grown in inoculated soils from 4 sites (A, B, C, D) and in 100% sterilized soil (control). Results of an analysis of variance (ANOVA) with factors “site” and “plot nested within site” are also depicted. An ANOVA followed by a Dunnett post-hoc test indicated that neither root nor shoot biomass of control plants significantly differed from the plants grown in inoculated soil

There was no effect of site on shoot biomass (ANOVA: F_(3,32)_ = 2.13, *P* = 0.115) but root biomass differed significantly between sites (ANOVA: F_(3,32)_ = 4.09, *P* = 0.025) (Fig. 5). After plant growth, soil characteristics in all pots were similar and did not differ between treatments (Supplementary Table 4), and there was no relationship between soil characteristics and metabolome composition (CoCa Test on all axes: trace = 0.0001, *P* = 0.757).

## Discussion

Our study shows that soil inoculation changed the metabolome of *J.vulgaris*. Further the metabolome alternations varied among the sites from which the inocula were collected. Moreover, metabolomes were more similar when plants were compared that had been grown with soil collected from different plots but from the same site than from plots that originated from different sites. We investigated the metabolomic changes with an untargeted metabolomics approach and then focused on the compounds that were related to the differences between plants grown in sterile and inoculated soils. Interestingly there were no distinct differences in metabolomes for all sites, but in plants in which metabolites were changed through inoculation, this consistently led to changes in the same compounds.

In accordance with our first hypothesis inoculation of sterilized soils with live soil lead to changes in the shoot metabolome of *J. vulgaris,* in particular in primary metabolites such as sugars. Other work has shown that phenolics and PA concentrations in *J. vulgaris* vary depending on the soil in which the plant was grown (Joosten et al. 2009; Wang et al. 2019b). With this study we now show that also other metabolites change upon inoculation and by not only targeting specific compounds or groups of compounds we can now provide the first fully untargeted metabolomics analysis of the response of *J. vulgaris* to soil inoculation. Our study provides evidence that metabolomes of plants can be modulated by inoculation of soils. The concentration of one PA decreased in almost all plants grown in soils inoculated with live soil compared to those grown in pure sterilized soil. An earlier study showed that damage at the roots or shoots of *J.vulgaris* leads to a decrease in concentrations and to changes in the composition of PAs (Kostenko et al., 2013). This suggests that certain PAs are involved in root defences upon attack and is probably related to the fact that certain PAs are synthesized in the roots (Hartmann 1999) and therefore more likely influenced by interactions in the soil than compounds which are synthetized in the shoots. Therefore, in our study the decrease of PA A in the shoots of the plants grown in inoculated soil could point at increased defence of the roots. Malic acid was also lower in plants grown in inoculated soils (A&C) than in plants grown in sterilized soil. Malic acid has many ecological functions. In root exudates it can attract beneficial rhizobacteria (Rudrappa et al. 2008) but also pests such as wireworms (*Agriotes* spp.) (Thorpe et al. 1947). Aboveground exogenous application of malic acid on shoots leads to increased chlorophyll contents (Darandeh and Hadavi 2012), however how changes in malic acid influence above ground interactions is not clear. All the other compounds which changed in concentrations are known to influence interactions with herbivores aboveground.

Inoculation with soil from certain sites, but not all sites, lead to metabolomic changes in the leaves of *J. vulgaris* when compared to the patterns observed in 100% sterilized soil. This may be relevant for our understanding of plant-insect interactions in the field for this species. In nature the microbial composition of soils changes at small spatial scales (Ettema and Wardle 2002; Fierer 2017) and our study now shows that such differences can potentially lead to changes in the metabolic characteristics of plants causing spatial heterogeneity in chemical composition among plants in the field. These metabolomic changes can influence the behaviour of enemies and beneficial organisms above and belowground in the field by attracting or deterring them (Van Dam et al. 1995; Vrieling et al. 1990, Kostenko and Bezemer, 2013). Hence, spatial variation in the composition of the microbiome in the soil may be one of the reasons for the often-unexplained chemical variation among plants in the field (Kostenko and Bezemer, 2013). However, this remains to be tested in more natural setups.

The concentrations of several compounds such as trehalose, tyrosine and an unknown PA (PA B) increased if *J. vulgaris* grew with inoculum from site A. Trehalose is involved in stress responses in plants and its effects can be either protective or adverse both in response to abiotic and biotic stress (Fernandez et al. 2010). Interestingly, trehalose can be produced by microorganisms such as endophytes and can change the plant’s ability to cope with stress (Vílchez et al. 2016). Therefore the higher concentrations of trehalose in plants grown with inocula from site A might hint at a specific community of endophytic bacteria that is transferred from the soil to the plant. The synthesis of phenolics, lignins and flavonoids in cell walls all require tyrosine as a precursor (Walling 2000) and therefore changes in tyrosine concentrations can have far reaching consequences for cell wall properties. Furthermore, insects with sclerotized cuticles, such as Coleoptera, require tyrosine for the synthesis of their cuticle (Andersen, 2010). Tyrosine is a limiting resource for insects with a sclerotized cuticle and changes in tyrosine content in the food source brought by changes in the soil therefore have the potential to influence the fitness related characteristics of herbivorous insects. We show that different parts of the metabolome of *J. vulgaris*, such as primary and secondary compounds can be influenced by the soil in which a plant is grown. These results emphasise the importance of untargeted metabolomic fingerprinting approaches to investigate the chemical response of plants to interactions with the soil rather than focusing on a few target compounds in the plant. Most studies, so far, have focused on secondary compounds however recently the importance of investigating the response of all plant compounds to abiotic and biotic factors is increasingly acknowledged (Peters et al. 2018). Not only can changes in primary compounds scale up to changes in secondary compounds over time, but these compounds themselves can also influence interactions of plants with organisms in their surroundings both above and belowground (Berenbaum 1995; Hervé and Erb 2019; Zhou et al. 2015).

All soils in which *J. vulgaris* was grown consisted of 90% sterilized bulk soil. This greatly reduced the potential effects of nutritional differences among soil inocula on the metabolome. This is confirmed as there were no differences in soil chemical characteristics among the soils in which *J. vulgaris* had grown. It is important to note that sterilized soil does not stay sterile and that microbiomes certainly were also present in pots with 100% sterilized soil. However other studies have shown that the composition of these soil microbiomes varies greatly from those in inoculated soils (e.g. Ma et al. 2018). Therefore, we propose that the differences that we observed in metabolome composition were caused by differences in soil microbial communities in the different inocula. Different microbial communities may vary in their direct effects on the plant (e.g. mutualists or pathogens) or indirectly affect the plant via influencing abiotic characteristics of the soil such as the nutrient availability or pH in the soil. Differences that we observed between metabolomes of plants grown in sterilized and live soil can be due to absence of specific (groups of) microbes in the sterilized soil. For example, the AMF *Rhizophagus irregularis,* can cause changes in the metabolome of *J. vulgaris* (Hill et al. 2018). In that study no changes were detected in the shoot metabolome but other studies with different plant species have shown that beneficial bacteria and AMF can influence foliar metabolomes (Schweiger et al. 2014; Zhou et al. 2018). Our results show that soils can influence the metabolome of plants and that these changes are probably caused by different microorganisms that are present in the different inocula. However, we can only speculate about the potential causes of these changes since we did not measure the microbial composition present in the soil.

Changes in plant metabolomes can arise from differences in the biomass of the plant (Lisec et al. 2008). In our study, we did not find a significant difference in shoot biomass among plants grown in inoculated soils from different sites. However, there was a strong relationship between plant shoot biomass and the leaf metabolome. All signals not associated to sugars were negatively or not correlated with shoot biomass. This can be due to several reasons. First there can be a dilution effect of all other compounds in the metabolome due to increased biomass. A higher photosynthetic activity can lead to a higher sugar content and the production of other compounds may lack behind. Second, plants that produced most biomass aboveground grew in 100% sterilized soil. Twenty to 40 % of a plant’s carbon fixed through photosynthesis is exuded into the soil by the roots (Badri and Vivanco 2009). We speculate that plants grown in 100% sterilized soil and with inocula from sites B, C and D may have spent less carbon for exudation to maintain their soil microbiome and might therefore have higher concentrations of sugars. This remains to be tested in future research. Interestingly, root biomass varied significantly depending on the origin of the inoculum. This indicates that inoculation, and presumably, differences in soil microbial communities impact root growth of *J. vulgaris* much more than shoot growth (Bezemer et al. 2013). But root biomass did not significantly explain variation of the metabolome. This shows that plant soil interactions which influence the root biomass without changing shoot biomass can still influence the metabolome of the shoots.

We found evidence for our third hypothesis that the dissimilarity in metabolome composition will depend on the spatial distance. The metabolomes of plants grown in soils inoculated with inocula collected from the same plot were more similar than those of plants that were grown with inocula from more distant sites. Therefore, our study provides some evidence for a spatial soil effect. We speculate that this is linked to higher similarity in the microbial community in the soil on plot level than at larger spatial scales, as shown previously in other studies (Brockett et al. 2012; Constancias et al. 2015; Oda et al. 2003; Wang et al. 2017; Xue et al. 2018). Previous research has highlighted that spatial heterogeneity in abiotic factors and spatial distance can explain the composition of microbial communities on local, regional and intercontinental scales (Hanson et al. 2012; Vos et al. 2013). With our study we show that the spatial distribution of soil can also cause spatial differences in the metabolomes of plants growing in these soils. Further studies should examine how spatial variation and the interplay of abiotic and biotic factors in the soil influence the chemistry of the plants grown in these soils in nature.

In conclusion we show that soil inoculation leads to changes in the composition of the leaf metabolome of *J .vulgaris*. In natural systems such differences could lead to variation in susceptibility to pathogens, and herbivores above and belowground and ultimately influence the abundance of these higher trophic levels.

## Electronic supplementary material

ESM 1 (DOCX 1030 kb)

## Data Availability

Data will be made available upon publication from Dryad Digital Repository (10.5061/dryad.p2ngf1vm5)
