# Supplementary material for: Soil Inoculation Alters Leaf Metabolic Profiles in Genetically Identical Plants
Source: J Chem Ecol. 2020 Feb 5;46(8):745–55. doi: 10.1007/s10886-020-01156-8 (PMC7429552; doi:10.1007/s10886-020-01156-8)

**Supplementary Material**

**SOIL INOCULATION ALTERS LEAF METABOLIC PROFILES IN GENETICALLY IDENTICAL PLANTS**

MARTINE HUBERTY*^1,2,3^, BEVERLY MARTIS^2^, JORIAN VAN KAMPEN^2^, YOUNG HAE CHOI^3,4^, KLAAS VRIELING^2^, PETER G. L. KLINKHAMER^2^, T. MARTIJN BEZEMER^1,2^

*^1^ Department of Terrestrial Ecology, Netherlands Institute of Ecology, Wageningen, The Netherlands*

*^2^ Plant Ecology and Phytochemistry, Institute of Biology, Leiden University, Leiden, The Netherlands*

*^3^ Natural Products Laboratory, Institute of Biology, Leiden University, Leiden, The Netherlands*

*^4^ College of Pharmacy, Kyung Hee University, Seoul, Republic of Korea*

ORCHID:MH: https://orcid.org/0000-0003-1609-1544,YHC: https://orcid.org/0000-0003-1484-1700, KV: https://orcid.org/0000-0001-6242-3808, PGLK: https://orcid.org/0000-0001-8650-2199, TMB: https://orcid.org/0000-0002-2878-3479

Author for correspondence:

Martine Huberty, E-mail: M.Huberty@nioo.knaw.nl, https://orcid.org/0000-0003-1609-1544

**Table S1** Results of permutational multivariate analysis of variance (PERMANOVA) testing the effect of inoculation with soil from four different sites (A, B, C, D) and plants grown in 100% sterilized soil, and shoot biomass on the metabolome of *Jacobaea vulgaris.* Presented are F-values with degrees of freedom (df), explained variance (R^2^) and P-values. Permutations were set to 999. Significant factors are indicated in bold

| Factor | F-value | R^2^ | P-value |
| --- | --- | --- | --- |
| **Site** | F_(4,59)_=3.57 | 0.14 | 0.002 |
| **Shoot biomass** | F_(1,59)_=18.46 | 0.19 | 0.001 |
| **Site * Shoot biomass** | F_(4,59)_=1.96 | 0.08 | 0.027 |

**Table S2** Results of a pairwise comparison of metabolomes with the function pairwiseadonis. Depicted are differences in the metabolome of *Jacobaea vulgaris* grown in inoculated soil from different sites (A, B, C, D) and in 100% sterilized soil (Control). Depicted are degrees of freedom (df), F values, explained variance (R^2^) and P-values (non-adjusted and adjusted). P-values were adjusted with FDR correction for multiple comparisons. Significant factors are indicated in bold

| Pair | | | df | F | R^2^ | P | Adjusted P |
| --- | --- | --- | --- | --- | --- | --- | --- |
| **A** | **vs** | **D** | **31** | **3.76** | **0.11** | **0.016** | **0.040** |
| **A** | **vs** | **C** | **31** | **4.26** | **0.12** | **0.004** | **0.037** |
| **A** | **vs** | **B** | **31** | **4.74** | **0.14** | **0.011** | **0.037** |
| **A** | **vs** | **Control** | **20** | **4.06** | **0.18** | **0.011** | **0.037** |
| D | vs | C | 31 | 3.11 | 0.09 | 0.039 | 0.078 |
| D | vs | B | 31 | 1.58 | 0.05 | 0.182 | 0.260 |
| D | vs | Control | 20 | 0.68 | 0.03 | 0.569 | 0.569 |
| C | vs | B | 31 | 0.75 | 0.02 | 0.532 | 0.569 |
| C | vs | Control | 20 | 2.62 | 0.12 | 0.054 | 0.090 |
| B | vs | Control | 20 | 0.96 | 0.05 | 0.392 | 0.490 |

**Table S3** Results of permutational multivariate analysis of variance (PERMANOVA) testing the effect of inoculation with soil from four different sites (A, B, C, D) and root biomass on the metabolome of *Jacobaea vulgaris.* Presented are F-values with degrees of freedom (df), explained variance (R^2^) and P-values. Permutations were set to 999. Significant factors are indicated in bold

| Factor | F-value | R^2^ | P-value |
| --- | --- | --- | --- |
| **Site** | **F_(3,56)_=3.13** | **0.13** | **0.005** |
| Root biomass | F_(1,56)_=2.69 | 0.04 | 0.054 |
| Site * Root biomass | F_(3,59)_=1.40 | 0.06 | 0.183 |

**Table S4** Mean concentrations of soil characteristics (±SE) for each plot (n = 2) measured after growth of *Jacobaea vulgaris.* Four out of eight plots were randomly selected for each site for the analysis. The differences between the soil characteristics were compared with analysis of variance (ANOVA) with fixed factor "soil" (A, B, C, D, Control (100% sterilized soil))*.* All chemicals were measured in mg/kg

| Site | | Plot | | Fe | K | Mg | NO_2_+NO_3_ | NH_4_-N | P | S | Zn | |
| --- | --- | --- | --- | --- | --- | --- | --- | --- | --- | --- | --- | --- |
| A | 1 | | 0.14±0.002 | | 9.35±3.15 | 29.05±0.65 | 5.04±0.19 | 1.01±0.01 | 3.11±0.47 | 3.63±0.30 | 0.63±0.23 | |
|  | 6 | | 0.17±0.013 | | 6.30±1.00 | 32.10±0.10 | 5.69±0.86 | 1.64±1.24 | 4.47±0.30 | 3.22±0.15 | 1.51±0.26 | |
|  | 7 | | 0.15±0.016 | | 7.65±1.55 | 30.50±0.50 | 4.92±1.49 | 1.23±0.73 | 3.45±0.32 | 3.32±0.53 | 0.86±0.19 | |
|  | 8 | | 0.14±0.007 | | 7.60±1.90 | 30.80±0.40 | 5.41±1.04 | 0.94±0.48 | 4.00±0.13 | 3.88±0.30 | 0.85±0.16 | |
|  |  | |  | |  |  |  |  |  |  |  | |
| B | 3 | | 0.15±0.012 | | 7.15±0.35 | 28.00±1.50 | 4.96±1.20 | 1.28±0.11 | 3.80±0.03 | 2.92±0.21 | 1.15±0.05 | |
|  | 5 | | 0.17±0.015 | | 10.55±1.25 | 32.55±0.35 | 3.40±1.52 | 1.89±0.44 | 3.12±0.21 | 3.12±0.38 | 0.82±0.13 | |
|  | 6 | | 0.17±0.034 | | 12.60±0.10 | 30.55±0.75 | 2.16±2.02 | 1.61±0.80 | 2.12±0.07 | 2.71±0.12 | 0.57±0.28 | |
|  | 8 | | 0.17±0.034 | | 8.25±2.35 | 33.00±3.20 | 5.48±1.23 | 1.04±0.73 | 2.92±0.68 | 3.58±0.99 | 1.04±0.60 | |
|  |  | |  | |  |  |  |  |  |  |  | |
| C | 4 | | 0.17±0.003 | | 8.75±2.35 | 31.60±0.20 | 3.63±2.54 | 1.17±0.53 | 3.70±0.31 | 3.21±0.44 | 1.39±0.14 | |
|  | 5 | | 0.16±0.005 | | 7.40±0.30 | 30.75±1.55 | 10.39±5.24 | 1.30±0.38 | 3.62±0.21 | 2.96±0.01 | 1.31±0.27 | |
|  | 7 | | 0.14±0.005 | | 6.05±0.45 | 27.55±3.25 | 5.20±0.48 | 1.18±0.78 | 3.42±0.91 | 2.81±0.35 | 1.04±0.31 | |
|  | 8 | | 0.14±0.016 | | 10.75±2.55 | 26.55±1.05 | 2.68±1.12 | 3.11±0.65 | 2.25±0.10 | 3.25±0.01 | 0.64±0.15 | |
|  |  | |  | |  |  |  |  |  |  |  | |
| D | 3 | | 0.17±0.012 | | 9.85±1.55 | 30.40±5.10 | 6.60±0.87 | 2.06±0.79 | 3.86±0.86 | 5.19±2.89 | 1.70±0.38 | |
|  | 4 | | 0.17±0.017 | | 6.20±0.60 | 32.05±0.85 | 4.67±1.09 | 0.78±0.24 | 3.59±0.55 | 3.40±0.53 | 1.27±0.23 | |
|  | 5 | | 0.21±0.017 | | 8.40±0.10 | 28.70±1.40 | 2.92±0.97 | 2.15±0.29 | 2.98±0.11 | 3.16±0.07 | 1.20±0.28 | |
|  | 8 | | 0.16±0.002 | | 8.35±0.75 | 27.90±0.80 | 8.69±6.34 | 2.24±0.99 | 3.29±0.09 | 3.32±0.16 | 1.04±0.22 | |
|  | | |  | |  |  |  |  |  |  |  |  |
| Control | | | 0.16±0.019 | | 6.70±0.32 | 31.58±1.09 | 5.74±0.52 | 1.51±0.48 | 3.88±0.34 | 3.48±0.37 | 1.06±0.34 |  |
|  | | |  | |  |  |  |  |  |  |  |  |
| ANOVA | | | F_(4,31)_=1.54 P=0.214 | | F_(4,31)_=1.29 P=0.295 | F_(4,31)_=0.86 P=0.499 | F_(4,31)_=0.39 P=0.817 | F_(4,31)_=0.51 P=0.728 | F_(4,31)_=1.69 P=0.177 | F_(4,31)_=0.75 P=0.566 | F_(4,31)_=0.98 P=0.430 |  |
|  | | |  | |  |  |  |  |  |  |  |  |

**Fig. S1** Non-metric multidimensional scaling (NMDS) of the metabolome of *Jacobaea vulgaris* grown with inocula collected from different sites (A, B, C, D) and in 100% sterilized soil using Bray-Curtis dissimilarities. The individual samples are coloured by shoot biomass. The stress is a measurement for the fit of the model and was 0.10

**
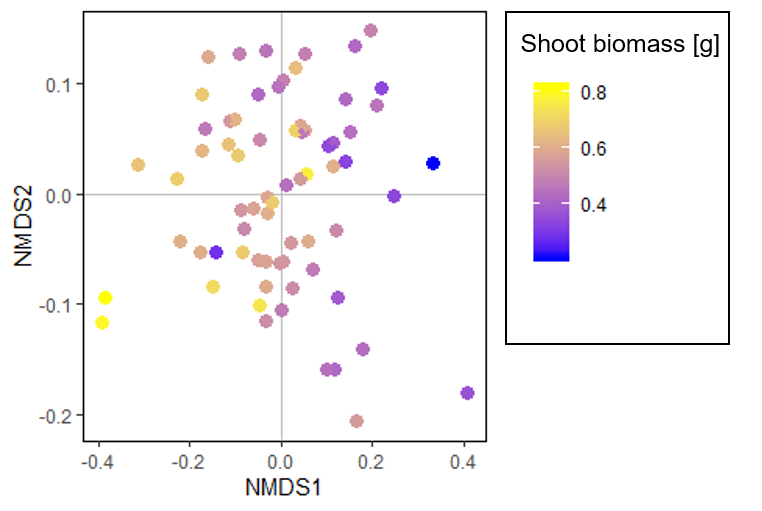
**

**Fig. S2** Negative logarithm of p-values from an analysis of variance (ANOVA) testing for differences in intensity of signals in each bucket (chemical shift) of the metabolome of *Jacobaea vulgaris* for (a) the site from which the soil inocula was collected from, (b) shoot biomass, and (c) and the interaction between site and shoot biomass. Grey dots represent signals for which P-values were smaller than 0.05 after false discovery correction (FDR). Putative identifications of significant signals are indicated in the panels. No description indicates that no specific compound could be assigned to this signal. The chemical shift of the signals that were associated to the different compounds were: trehalose 5.16 ppm, tyrosine 3.16 and 3.9 ppm , glucose 3.2 ppm, pyrrolizidine alkaloid A (PA A) 2.48 ppm, malic acid 2.72 ppm, alanine 1.48 ppm, raffinose 5.44 ppm, adenosine 8.24 ppm, formic acid 8.44 ppm, mannitol 3.88 ppm, glutamate 2.36 ppm glutamine 2.4 ppm. The circle in (b) describes signals with a chemical shift in the region which is associated with signals from sugars


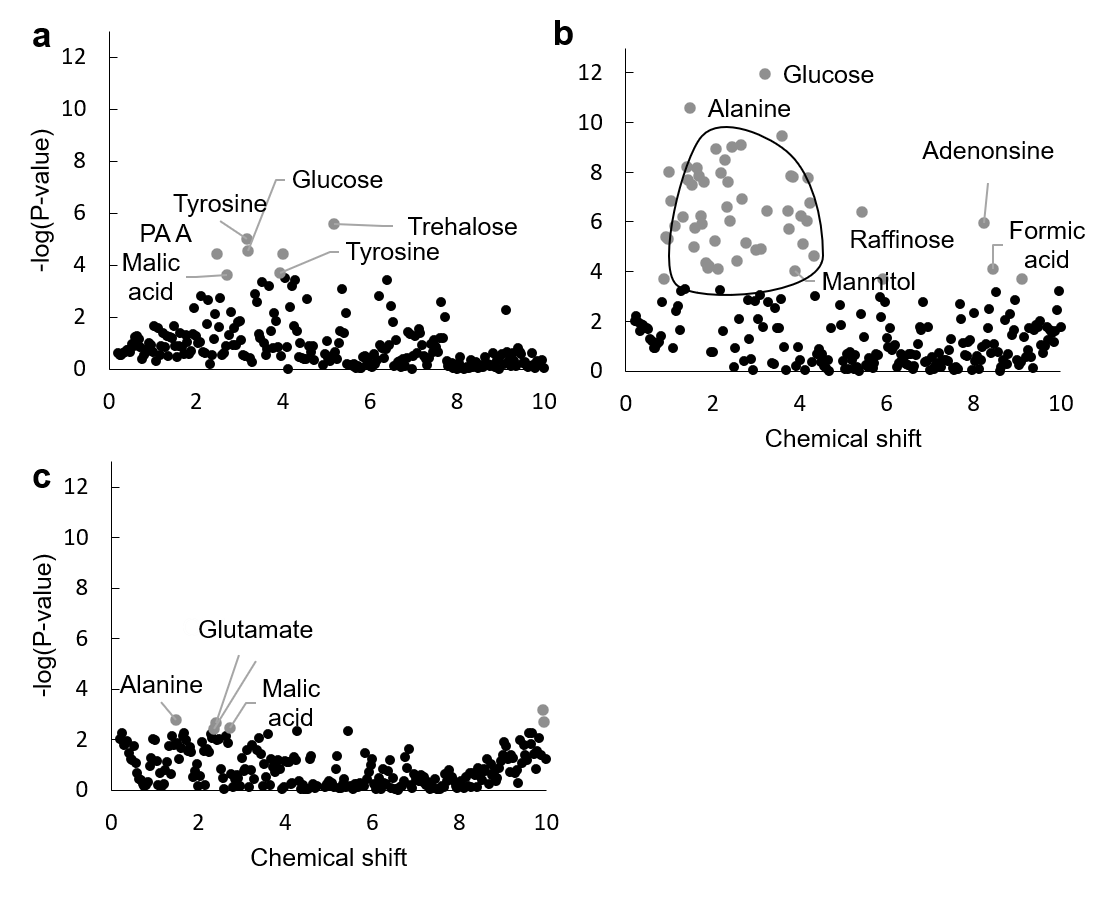


**Fig. S3** Pearson correlation coefficient between shoot biomass of *Jacobaea vulgaris* and the intensity of the signals of the different chemical shifts (buckets). Grey dots indicate significant correlations after false discovery correction (FDR). Chemical shifts of buckets are indicated by numbers. A description of compounds which are often associated to a specific chemical shift is also presented. Two different pyrrolizidine alkaloids are abbreviated with PA A respectively PA B. Abbreviations for amino acids are: LEU= Leucine, ALA=Alanine, THR=Threonine, GLU=Glutamic acid, TYR=Tyrosine, HIS=Histidine.


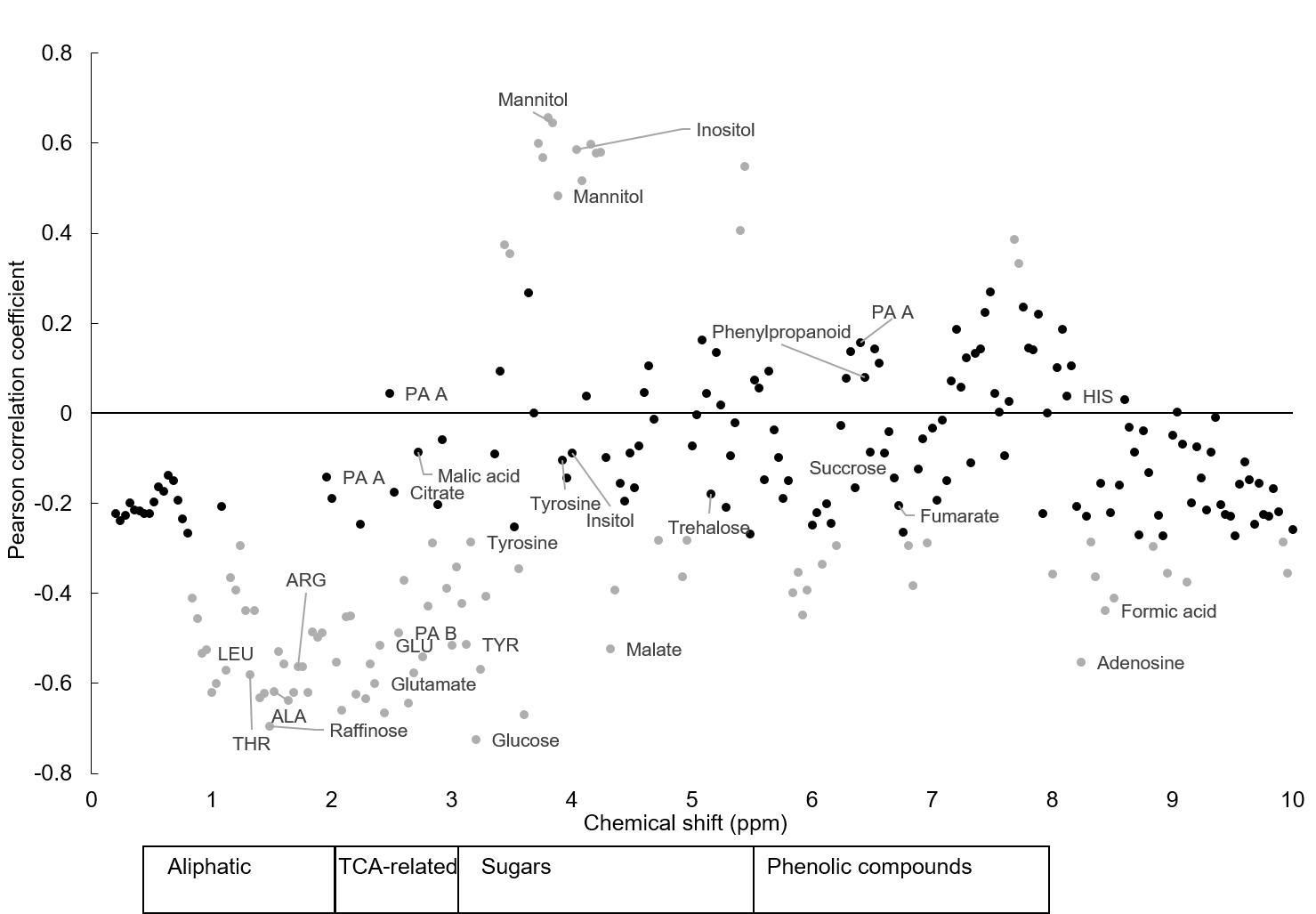


**Fig.S4** Volcano plots depicting metabolomic differences of *Jacobaea vulgaris* grown in inoculated soil from four sites (A, B, C and D) compared to 100% sterilized soil. Colour dots in the volcano plot outside the cut-off lines depict signals with a fold-change (FC) >2 and P < 0.1 based on a t-test (unadjusted). The signals within these buckets were putatively assigned to compounds with signals identified as pyrrolizidine alkaloids A (PA A) at 2.48 and 6.52 ppm, malic acid at 2.72 ppm, trehalose at 5.16 ppm, tyrosine at 3.16 ppm and pyrrolizidine alkaloid B (PA B) at 2.56 ppm. Signals which could not be assigned to a specific metabolite were found at 6.48, 6.52 ppm and 0.4-0.6 ppm. Negative fold-changes represent signals that show a higher intensity in plants grown in 100% sterilized soil (left side) than in inoculated soils. Positive fold-changes represent signals that show a higher intensity in plants grown in the inoculated soil compared to the 100% sterilized soil


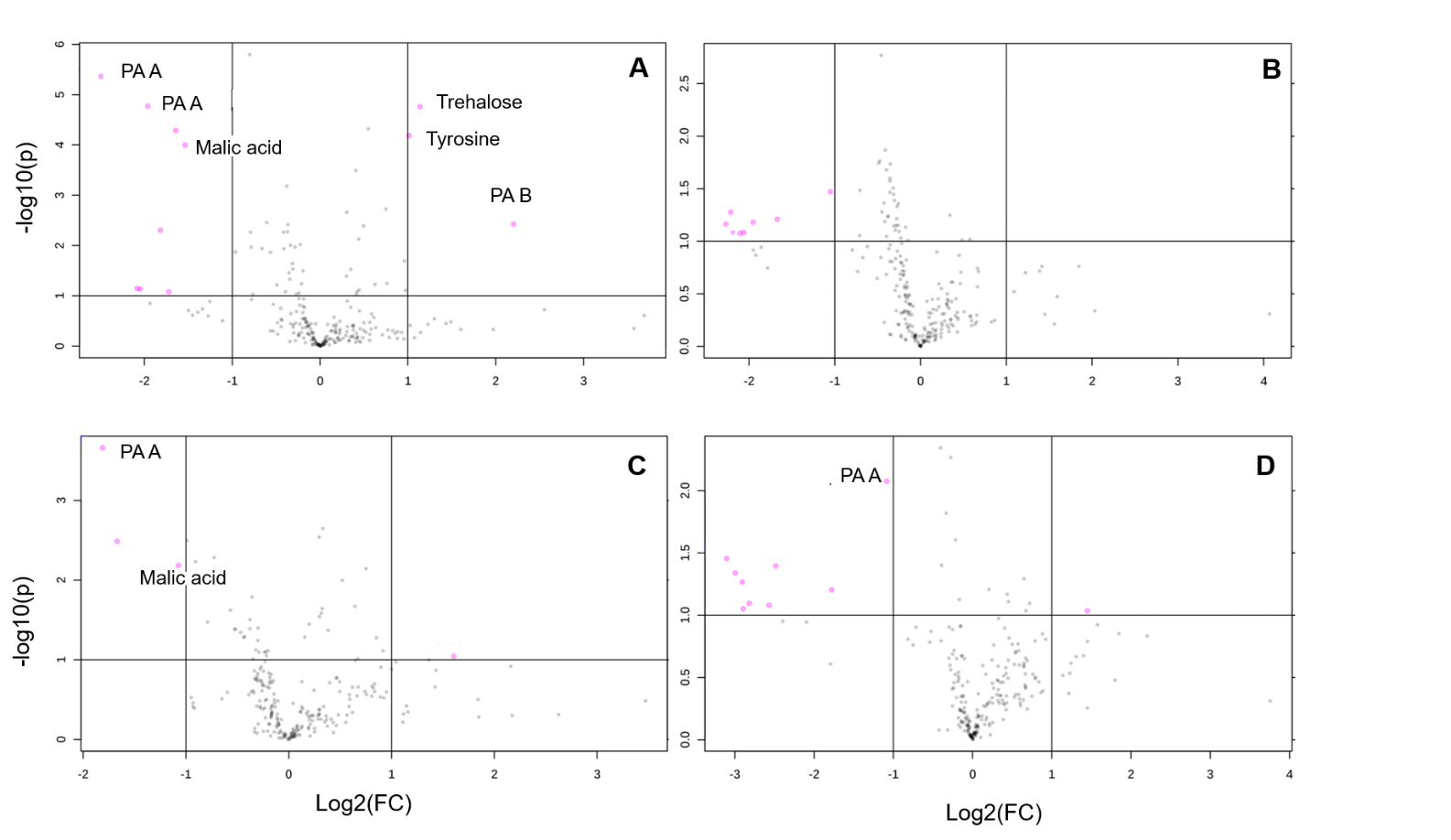


**Fig. S5** Mean Shannon evenness (±SE) of *Jacobaea vulgaris* leaf metabolomes of plants grown with inocula from 4 different sites (A, B, C, D) and in 100% sterilized soil with shoot biomass as covariate. The results of an analysis of variance (ANOVA) testing for the effects of site, plots nested within site, and shoot biomass is also presented. For this analysis the 100% sterilised soil treatment was removed from the dataset. A Dunnett post-hoc test did not reveal significant differences among bars


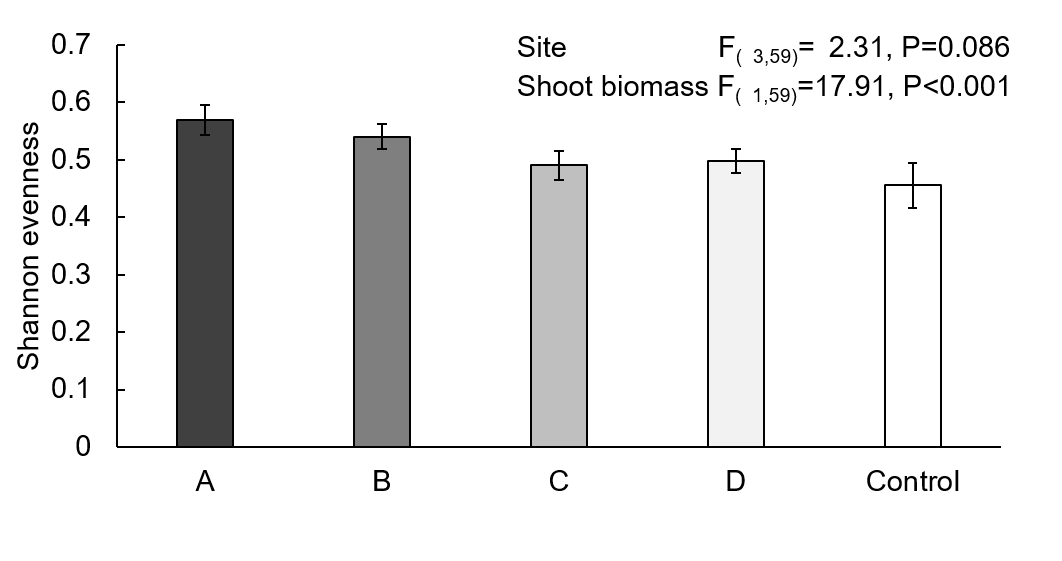

Supplement: Supplementary file 1 — (DOCX 1030 kb) [file 10886_2020_1156_MOESM1_ESM.docx]
